# Lebanese students’ motivation in medical school: does it change throughout the years? A cross-sectional study

**DOI:** 10.1186/s12909-020-02011-w

**Published:** 2020-03-31

**Authors:** Anne-Sophie Sarkis, Souheil Hallit, Aline Hajj, Anthony Kechichian, Dolla Karam Sarkis, Antoine Sarkis, Eliane Nasser Ayoub

**Affiliations:** 1 Faculty of Medicine, Saint-Joseph University, Beirut, Lebanon; 2 Faculty of Medicine and Medical Sciences, Holy Spirit University of Kaslik (USEK), Jounieh, Lebanon; 3 INSPECT-LB : Institut National de Santé Publique, Epidémiologie Clinique et Toxicologie, Beirut, Lebanon; 4 Faculty of Pharmacy, Saint-Joseph University, Beirut, Lebanon; 5 Laboratory of Pharmacology, Clinical Pharmacy and Quality Control of Drugs, Faculty of Pharmacy, Pôle Technologie-Santé (PTS), Faculty of Pharmacy, Saint-Joseph University, Beirut, 1107 2180 Lebanon; 6 Laboratory of Molecular Microbiology, Faculty of Pharmacy, Saint-Joseph University, Pôle Technologie-Santé (PTS), Faculty of Pharmacy, Saint-Joseph University, Beirut, 1107 2180 Lebanon; 7 Department of Cardiology, Hôtel-Dieu de France Hospital, Beirut, Lebanon; 8 Department of Anesthesiology and reanimation, Hôtel-Dieu de France Hospital, Beirut, Lebanon

**Keywords:** Amotivation, Extrinsic motivation, Instrinsic motivation, Medicine, Students

## Abstract

**Background:**

Students entering medical school are driven by different types of motivation: autonomous motivation, controlled motivation, or amotivation. Motivation types can influence students’ performance, outcome and well-being. To our knowledge, this topic has never been studied in Lebanese medical students. This study aims to identify students’ motivation types in the first 5 years of medical school at two Lebanese universities (USJ and USEK). It also aims to determine the predominant motivation type of the whole sample. Results may be the first step towards raising awareness about this topic and implementing actions that enhance autonomous motivation.

**Methods:**

A cross-sectional study was performed between January and June 2017. A questionnaire was sent to medical students by e-mail. The students’ academic motivation was assessed using the Academic Motivation Scale.

**Results:**

A higher mean autonomous motivation score was found in each academic year, as compared to the mean controlled motivation and amotivation scores. The highest mean autonomous motivation score was seen among second year students, whereas the lowest score was noted in fifth year students. The highest scores for controlled motivation and amotivation belonged to the fourth-year students, and the lowest to the first-year students. Students who were still satisfied with medical studies had a higher autonomous motivation score. Finally, USJ students who were satisfied with their second year training had a higher mean autonomous motivation score than those who were not.

**Conclusion:**

This study showed high levels of autonomous motivation in the first five years of medical school. Autonomous motivation was the predominant type in the whole sample. The highest scores of controlled motivation and amotivation were noted in the fourth year. Moreover, high levels of self-determination were seen in students who enjoyed their early contacts with patients through trainings. Actions should be implemented in medical schools to enhance and maintain autonomous motivation, and consequently students’ outcome and health-care quality.

## Background

**Fig. 1:**
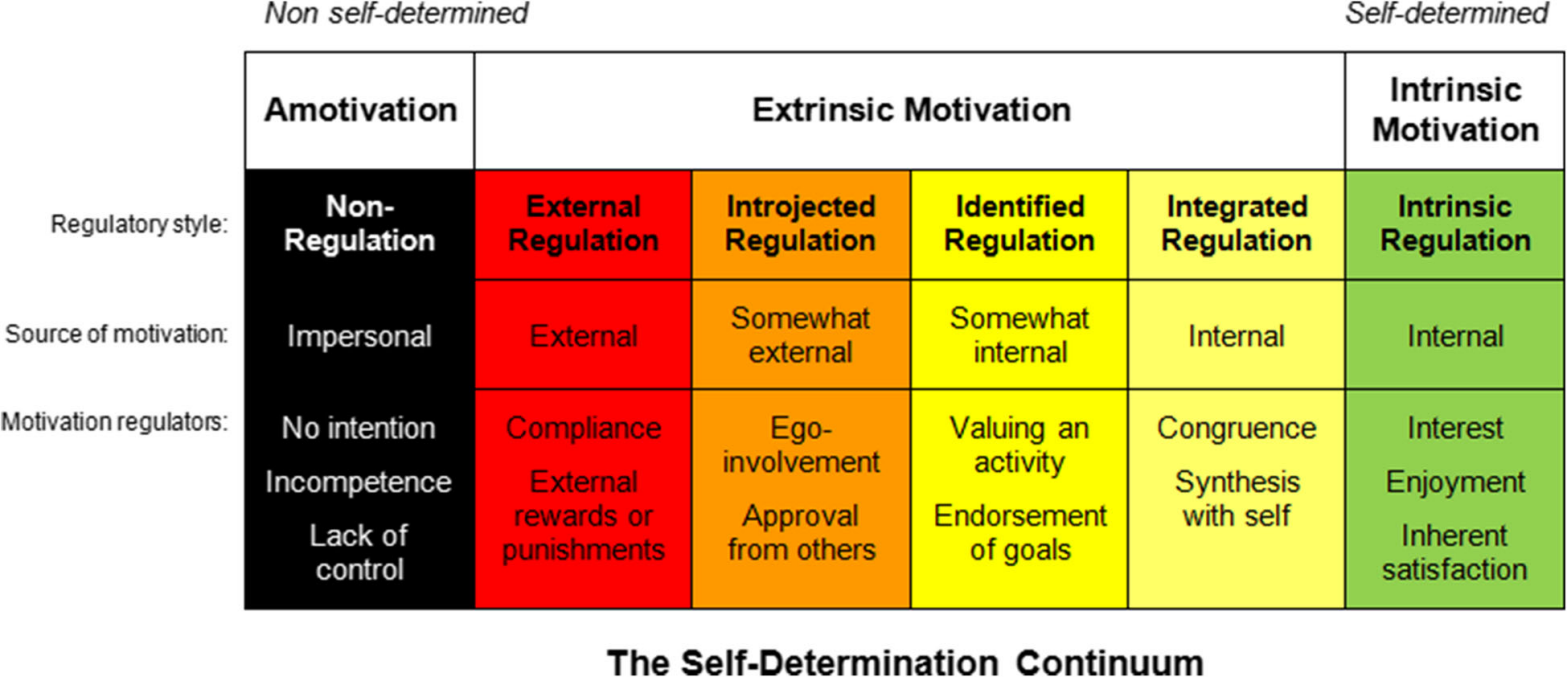
The Self-Determination Continuum **(**https://learningsnippets.files.wordpress.com/2013/10/sdt-continuum.png)

The concept of motivation has become an important research subject in the field of education. “Motivation” can be defined as “a reason for acting or behaving in a particular way” [1]. A widely-used approach to motivation is the theory of Deci and Ryan [2]. This theory was elaborated in the 1980s, and is based on the level of self-determination. It states that motivation can be internally or externally generated, with a higher or lower level of self-determination, respectively. A motivation type can be identified for each student (Fig. 1). A student with genuine interest in medicine has an intrinsic motivation. Someone studying medicine because of external factors has an extrinsic motivation, which can be further sub-classified: external motivation with identified regulation (e.g., studying medicine to reach a personal goal, with little interest in medicine itself), introjected regulation (e.g., behavior influenced by social expectations or by internalized controllers), and external regulation (e.g., behavior influenced by the system of punishment and reward). Finally, if students have no interest at all in medicine, they are classified as amotivated. Intrinsic motivation and extrinsic motivation with identified regulation are considered “autonomous motivation”, whereas extrinsic motivation with introjected and external regulations are considered “controlled motivation” [3].

Types of motivation are key elements in any educational system as they shape students’ behavior, along with their consequent performance and outcome [4]. According to the self-determination theory, intrinsic motivation and autonomous forms of self-regulation are desirable since they have been associated with deep learning, better performance and well-being [5]. Three basic psychological needs have to be satisfied in order to achieve intrinsic motivation: the needs for autonomy, competence and relatedness. Autonomy refers to making decisions by one’s own will, based on their needs and values. Competence refers to the desire of feeling capable of performing a determined task. As for relatedness, it is the need for belongingness or connectedness with a significant community [4, 6–9]. Thus, several pedagogic strategies that stimulate internalization of motivation and autonomous types of motivation have been recently evaluated [10].

Previous studies have associated motivation types to different factors, including gender, academic year, and learning methods. Actually, studies have shown that intrinsic motivation is more prone to decline with the years of medical school [11]. Sobral’s [12] and Kusurkar’s [13] studies revealed that girls are more intrinsically motivated than boys. Finally, students who enjoy their clinical trainings have higher levels of self-determination [14].

To the best of our knowledge, no previous studies regarding motivation types in medical students have been previously conducted in Lebanon. The current study attempts to test and compare the case of medical students in Lebanon to results of studies conducted in other countries and, thus, bringing awareness to the Lebanese medical field on the topic of students’ motivation. Therefore, we decided to study the motivation types of students at the Faculty of Medicine of two different Lebanese universities: Saint-Joseph University of Beirut (USJ), and the Holy Spirit University of Kaslik (USEK). These two faculties follow the European Credit Transfer System (ECTS). The Faculty of Medicine of USJ has a larger number of students and more trainings during the curriculum, compared to that of USEK. At USJ, students have a nursing training in second year, a physical examination training in third year, and observerships in fourth and fifth years. At USEK, they have a nursing training in third year, and observerships in fourth and fifth years.

The primary objective of this reported study was to identify the types of students’ motivation across the first 5 years of the medical program at USJ and USEK, and determine the predominant type in the whole sample. The secondary objective was to identify variables that affect these motivation types. We hypothesized that autonomous motivation should increase with the years of the curriculum, since students get closer to practice, and that it should be the predominant type in the whole sample.

We hope this study helps create awareness for motivation types in the Lebanese medical system. It may reveal the variables that could be modified in order to enhance self-determination. It can benefit teachers, who could implement actions to support students’ autonomous motivation [5]. Additionally, it can benefit students, who will be aware of their motives and types, and enhance health care delivery.

## Methods

### Study design

A cross-sectional study was conducted to focus on types of motivation among medical students in Lebanon.

### Setting

The study was performed between January and June 2017, in the faculties of medicine at USJ and USEK. A list of enrolled students was provided by the administration of both faculties. USJ students were first approached in January 2017. USEK students participated in the study later, in June 2017.

### Participants

All 581 students from the first 5 years of the medical program were targeted (431 from USJ, 150 from USEK).

### Questionnaire

The questionnaire used in this study was adapted from the Academic Motivation Scale (AMS). We adapted each item of the questionnaire to the context of the medical field. The questionnaire was accessed online using two links, one for USJ and one for USEK. Those links were sent via emails to all medical students at participating universities. Reminders to answer were sent every 2 weeks for 6 weeks. There were no exclusion criteria. Each student was allowed to fill the questionnaire once.

In the first part of the survey, students were asked to enter their socio-demographic details (age, gender, marital status, year of study), and respond to a questionnaire on whether they belonged to a medical family or not, whether they lived with their parents or not, the reason for choosing a medical program, and the degree of satisfaction with their choice. The second part of the survey aimed at assessing the students’ type of motivation. The latter was studied using the Academic Motivation Scale in Education-University Studies (AMS), developed and validated in Quebec [11, 15]. This instrument was developed based on the theory of self-determination of Deci and Ryan [4, 11, 12]. It adds three subscales to intrinsic motivation, thus encompassing seven types of motivation: intrinsic motivation to knowledge (MICO), intrinsic motivation for accomplishment (MIAC), intrinsic motivation to stimulation (MIST), extrinsic motivation by identified regulation (MEID), extrinsic motivation by introjected regulation (MEIN), external extrinsic motivation (ERM), amotivation (AMOT). The questionnaire includes 28 items, grouped into those seven types. For each of the 28 items, the student had to specify to which extent the proposed statement is a reason for continuing medical studies on a five-point Likert scale, ranging from “strongly disagree” to “strongly agree”. Questions related to trainings of students during the first 5 years were also incorporated in the questionnaire. Students were asked about their satisfaction with the different trainings, and whether they wished to have more training hours.

### Statistical analysis

The collected data were analyzed using Statistical Package for Social Sciences (SPSS) software, version 23. The frequency of the quantitative variables was reported as the mean ± standard deviation, while the frequency of the qualitative variables was expressed in frequency and percentages. The Chi-2 test was used for dichotomous or multinomial qualitative variables. To confirm the validity of the questionnaire of students’ motivation types in the Lebanese population, a factor analysis was launched respectively for the questions of each type, using the principal component analysis technique with a Promax rotation since the extracted factors were found to be significantly correlated. The measurement of Kaiser-Meyer-Olkin sampling ability and the Bartlett sphericity test were found to be adequate. The number of factors retained corresponded to Eigen values ​​greater than one. In addition, reliability of the scale’s items was assessed by Cronbach’s alpha values. A *p* < 0.05 was considered significant.

## Results

Out of 581 questioned students, 206 (35.5%) filled the questionnaire. The sociodemographic and other characteristics of the participants are summarized in Table 1.

**Table 1:** Sociodemographic and other characteristics of the sample (*N* = 206)

	USJ (***N*** = 145)	USEK (***N*** = 61)	***p***-value
**Year of study**			**0.035**
1st year	**50** (34.5%)	**13** (21.3%)	
2nd year	15 (10.3%)	11 (18%)	
3rd year	**21** (14.5%)	**13** (21.3%)	
4th year	13 (9%)	11 (18%)	
5th year	**46** (31.7%)	**13** (21.3%)	
**Repeating the academic year**			0.526
No	**144** (99.3%)	**60** (98.4%)	
Yes	1 (0.7%)	1 (1.6%)	
**Gender**			0.635
Male	59 (40.7%)	27 (44.3%)	
Female	**86** (59.3%)	**34** (55.7%)	
**Marital status**			
Single	**144** (100%)	**61** (100%)	–
**Belonging to a medical family**			0.742
No	**108** (74.5%)	**46** (76.7%)	
Yes	37 (25.5%)	14 (23.3%)	
**Living with parents**			**0.043**
No	18 (12.4%)	2 (3.3%)	
Yes	**127** (87.6%)	**59** (96.7%)	
**Reason to be a doctor**			
Vocation and passion for medicine	**115** (79.3%)	**52** (85.2%)	0.321
Familial pressure	7 (4.8%)	4 (6.6%)	0.614
It’s a business that earns money	37 (25.5%)	15 (24.6%)	**< 0.001**
For the benefits that this profession brings, especially in Lebanon	45 (31%)	11 (18%)	0.056
It’s a liberal job	**77** (53.1%)	11 (18%)	**< 0.001**
For the humanitarian side of the profession	**105** (72.4%)	**37** (60.7%)	0.096
**Satisfaction with choice of medical studies**			
Yes	121 (84%)	54 (88.5%)	0.405
**Choice of specialty fixed from the beginning**			
Yes	42 (29%)	21 (34.4%)	0.437
**Choice of specialty modified during your medical studies**			
Yes	85 (60.3%)	39 (63.9%)	0.625
**Choice of specialty**			0.594
Medical specialty	63 (43.4%)	22 (36.1%)	
Surgery	42 (29%)	21 (34.4%)	
Others	40 (27.6%)	18 (29.5%)	

### Factor analysis

Among all the questions asked in the questionnaire, all variables, except question 7, could be extracted from the list during the factor analysis, since none of the questions were strongly correlated with another question (*r* > 0.9), had a low load factor (< 0.3) or a low community level (< 0.3). It is of note that question 7 was removed from the analysis due to a low communality < 0.3.

The factor analysis for the motivation type questionnaire for medical students was carried out on the entire sample (*n* = 206). The questionnaire elements converged on a solution of three factors, explaining a total of 81.51% of the variance (factor 1: items related to autonomous motivation; factor 2: items related to controlled motivation; factor 3: items related to amotivation). A Kaiser-Meyer-Olkin measurement of sampling adequacy of 0.975 was found, with a significant Bartlett’s test of sphericity (*p* < 0.001). In addition, a high Cronbach alpha was found for the whole scale (0.984) (Table 2).

**Table 2:** Factor analysis of the Academic Motivation Scale at the faculties of medicine of USJ and USEK

Factor 1	Item number	Loading on factor
***Why do you go to medical school?***		
For the pleasure that I experience when I discover the theories of famous researchers.	11	1.044
For the pleasure that I experience when I feel completely absorbed by medical studies and sciences.	18	1.035
For the pleasure I experience while surpassing myself in my medical studies.	6	.942
For the pleasure that I experience in broadening my knowledge about subjects which appeal to me.	16	.911
For the pleasure I experience when I discover new things in medicine that I have never seen before.	9	.908
For the pleasure that I experience while I am surpassing myself in one of my personal accomplishments.	13	.881
Because I experience pleasure and satisfaction while learning new things related to the medical field.	2	.872
For the “high” feeling that I experience while learning about breakthroughs in the medical field.	25	.858
Because my medical studies allow me to continue to learn about many things that interest me.	23	.858
For the intense feelings I experience when I am communicating my own ideas to others about a medical subject.	4	.783
Because I think that a college education will help me better prepare for the career I have chosen.	3	.766
For the satisfaction I feel when I am in the process of accomplishing difficult academic activities.	20	.749
Because medical school allows me to experience a personal satisfaction in my quest for excellence in my studies.	27	.683
Because eventually it will enable me to enter the job market in a field that I like.	10	.664
Because this will help me make a better choice regarding my career orientation.	17	.529
Because I believe that a few additional years of medical education will improve my competence as a worker.	24	.489
**Factor 2**		
In order to have a better salary later on.	22	.932
Because I want to have “the good life” later on.	15	.903
In order to obtain a more prestigious job later on.	8	.862
Because with only a high-school degree I would not find a high-paying job later on.	1	.724
To show myself that I am an intelligent person.	21	.683
Because I want to show myself that I can succeed in my medical studies.	28	.680
Because of the fact that when I succeed in medical school I feel important.	14	.546
**Factor 3**		
I don’t know; I can’t understand what I am doing in medical school.	26	.960
Honestly, I don’t know; I really feel that I am wasting my time in medical school.	5	.913
I can’t see why I go to medical school and frankly, I couldn’t care less.	19	.839
I once had good reasons for going to medical school; however, now I wonder whether I should continue.	12	.792

### Bivariate analysis

The total scores of autonomous motivation, controlled motivation and amotivation were computed based on the factor analysis results by adding the answers of the questions that constitute each factor. The average score was calculated by dividing the total score by the number of questions forming each factor. The results showed that the mean autonomous motivation score was 3.04 ± 0.94, whereas controlled motivation and amotivation scores were respectively 2.55 ± 1.02 and 1.64 ± 079.

In order to evaluate the variables that affect types of motivation, bivariate analyses have been performed. Results are summarized in Table 3. In the studied sample, a higher mean autonomous motivation score was found in each academic year, in comparison to mean controlled motivation and amotivation scores. The highest score for autonomous motivation (3.42 ± 0.92, *p* < 0.001) belongs to the second-year students, and the lowest score for the same (2.74 ± 0.80) to the fifth-year students. The highest scores for controlled motivation (3.06 ± 1.20, *p* < 0.001) and amotivation (2.12 ± 0.96, *p* < 0.001) belong to the fourth-year students. However, the lowest score of controlled motivation and amotivation (respectively 2.30 ± 0.92 and 1.38 ± 0.61) belongs to the first-year students.

**Table 3:** Bivariate analysis of the different scores with the sociodemographic variables among the whole sample

Variable / scores	Autonomous Motivation	Controlled Motivation	Amotivation
**Gender**			
Male	3.04 ± 0.92	2.65 ± 1.00	1.77 ± 0.93
Female	3.04 ± 0.96	2.47 ± 1.03	1.55 ± 0.66
*p*-value	0.995	0.247	0.076
**Year of study**			
1st year	2.88 ± 0.86	2.30 ± 0.92	1.38 ± 0.61
2nd year	3.42 ± 0.92	2.81 ± 1.21	1.89 ± 0.73
3rd year	3.32 ± 1.02	2.71 ± 1.14	1.58 ± 0.68
4th year	3.41 ± 1.09	3.06 ± 1.20	2.12 ± 0.96
5th year	2.74 ± 0.80	2.38 ± 0.75	1.65 ± 0.86
*p*-value	**< 0.001**	**< 0.001**	**< 0.001**
**Doctor in the family**			
No	3.05 ± 0.92	2.54 ± 0.99	1.70 ± 0.83
Yes	2.97 ± 0.99	2.53 ± 1.09	1.45 ± 0.63
*p*-value	0.610	0.964	**0.036**
**Living with parents**			
No	2.62 ± 0.70	2.15 ± 0.62	1.45 ± 0.61
Yes	3.09 ± 0.96	2.59 ± 1.04	1.66 ± 0.81
*p*-value	**0.018**	**0.016**	0.311
**Choice of being a doctor by vocation**			
No	2.70 ± 0.99	2.49 ± 1.01	1.86 ± 0.85
Yes	3.12 ± 0.92	2.56 ± 1.02	1.59 ± 0.77
*p*-value	**0.017**	0.696	0.072
**Familial pressure**			
No	3.04 ± 0.93	2.53 ± 1.02	1.63 ± 0.80
Yes	3.03 ± 1.21	2.86 ± 1.08	1.82 ± 0.65
*p*-value	0.983	0.327	0.450
**Business that earns money**			
No	3.02 ± 0.92	2.42 ± 0.97	1.59 ± 0.77
Yes	3.10 ± 1.01	2.93 ± 1.06	1.78 ± 0.83
*p*-value	0.653	**0.003**	0.164
**Benefits the profession brings**			
No	3.11 ± 0.94	2.50 ± 1.03	1.65 ± 0.82
Yes	2.83 ± 0.93	2.69 ± 0.98	1.62 ± 0.70
*p*-value	0.075	0.269	0.831
**Liberal job**			
No	3.26 ± 0.99	2.64 ± 1.10	1.75 ± 0.81
Yes	2.75 ± 0.77	2.42 ± 0.88	1.48 ± 0.74
*p*-value	**< 0.001**	0.139	**0.026**
**Humanitarian side**			
No	3.14 ± 1.01	2.69 ± 1.07	1.62 ± 0.76
Yes	3.00 ± 0.91	2.48 ± 0.99	1.65 ± 0.81
*p*-value	0.367	0.209	0.794
**Still satisfied from your choice**			
No	2.55 ± 0.91	2.45 ± 0.79	2.08 ± 0.99
Yes	3.13 ± 0.92	2.57 ± 1.06	1.56 ± 0.73
*p*-value	**0.004**	0.584	**0.002**
**Choice of specialty fixed from the beginning**			
No	2.96 ± 0.89	2.50 ± 0.99	1.60 ± 0.78
Yes	3.24 ± 1.03	2.67 ± 1.06	1.74 ± 0.80
*p*-value	0.086	0.311	0.299
**Choice of specialty changed during studies**			
No	3.10 ± 0.92	2.49 ± 0.96	1.68 ± 0.81
Yes	3.02 ± 0.97	2.61 ± 1.06	1.63 ± 0.79
*p*-value	0.605	0.433	0.682
**University**			
USJ	2.49 ± 0.39	2.02 ± 0.57	1.26 ± 0.48
USEK	4.26 ± 0.59	3.72 ± 0.79	2.48 ± 0.68
*p*-value	**< 0.001**	**< 0.001**	**< 0.001**

Post-hoc analysis for the year of study: Autonomous motivation score (2nd vs 5th year *p* = 0.049; 4th vs 5th year *p* = 0.049); Controlled motivation: 1st vs 4th year *p* = 0.03; amotivation 1st vs 4th year *p* = 0.001

As for score comparison within other variables, a higher mean autonomous motivation score was found in students who lived with their parents (3.09 ± 0.96, *p* = 0.018), who chose to be a doctor because it was their vocation (3.12 ± 0.92, *p* = 0.017), who did not choose to be a doctor for the liberal side of the job (3.26 ± 0.99, *p* < 0.001), and who are still satisfied with their choice of going to medical school (3.13 ± 0.92, *p* = 0.004).

Additionally, a higher mean controlled motivation score was found in students who lived with their parents (2.59 ± 1.04, *p* = 0.016), and in those who chose to be a doctor because they considered it a business that earns money (2.93 ± 1.06, *p* = 0.003).

Finally, a higher amotivation score was found in medical students who did not have a doctor in their family (1.70 ± 0.83, *p* = 0.036), and in those who were not satisfied anymore with their choice of going to medical school (2.08 ± 0.99, *p* = 0.002).

We also studied the correlation between the students’ trainings and their motivation type (Table 4). We noticed that at USJ, students who were satisfied with their second-year training scored significantly higher on autonomous motivation (2.54 ± 0.32, *p* = 0.027) than students who were not.

**Table 4:** Trainings satisfaction and motivation scores between both universities

	Autonomous Motivation	Controlled motivation	Amotivation
**USJ**			
**2nd year training**			
No	2.32 ± 0.46	2.08 ± 0.54	1.33 ± 0.46
Yes	2.54 ± 0.32	2.01 ± 0.63	1.26 ± 0.52
*p*-value	**0.027**	0.638	0.538
**3rd year training**			
No	2.41 ± 0.38	2.11 ± 0.58	1.25 ± 0.42
Yes	2.43 ± 0.42	2.05 ± 0.56	1.35 ± 0.51
*p*-value	0.827	0.661	0.439
**4th year training**			
No	2.24 ± 0.47	2.11 ± 0.58	1.46 ± 0.57
Yes	2.45 ± 0.36	2.14 ± 0.56	1.23 ± 0.37
*p*-value	0.135	0.878	0.147
**5th year training**			
No	2.23 ± 0.41	2.21 ± 0.50	1.39 ± 0.55
Yes	2.46 ± 0.39	2.09 ± 0.58	1.26 ± 0.40
*p*-value	0.108	0.529	0.387
**USEK**			
**3rd year training**			
No	4.19 ± 1.34	3.76 ± 1.28	3.50 ± 1.30
Yes	4.20 ± 0.26	3.41 ± 0.58	2.57 ± 0.61
*p*-value	0.988	0.689	0.088
**4th year training**			
No	3.73 ± 1.03	3.38 ± 1.14	3.41 ± 1.23
Yes	4.26 ± 0.23	3.54 ± 0.56	2.44 ± 0.43
*p*-value	0.465	0.747	0.304
**5th year training**			
No	3.95 ± 0.67	3.45 ± 0.76	2.83 ± 1.04
Yes	4.08 ± 0.26	3.08 ± 0.41	2.95 ± 0.69
*p*-value	0.701	0.360	0.836

## Discussion

Medical students’ life can be very challenging across the years, and “motivation” may go through ups and downs. Motivation types may play a significant role throughout these tough years, modulating students’ ambition and decisions [16–18]. We therefore conducted this study to identify the motivation types of Lebanese medical students gathered from two faculties. At the level of the whole sample, we were expecting autonomous motivation to increase over the years, as internship gets closer. Surprisingly, our main finding was that autonomous motivation actually remained predominant in all academic years. However, its highest level was observed in the second year, and its lowest level in the fifth year. The highest scores of controlled motivation and amotivation were observed in fourth-year students, and their lowest scores in first-year students. A study conducted in Brazil also showed a higher level of autonomous motivation in initial semesters of medical school (pre-clinical phase). However, it also showed higher levels of amotivation and extrinsic motivation with external regulation at later stages of medical school (clinical phase) [19]. This difference in motivation was attributed to the impact of learning environment, curriculum and medical school strategies: students have inherent characteristics when they enter medical school, but can become less intrinsically stimulated if courses are too theoretical and lack clinical contextualization [20]. Additionally, a study published in 2013 [11] described a decline in “idealism” (empathy and idealistic motivations) in fourth- and fifth-year medical students (“*first-year medical student MS1”* and “*second-year medical student MS2*”); it showed a shift of motives towards lifestyle, money, career and prestige [11]. Although “idealism” and “motivation” are two different concepts, the shift of motives described in the study refers to variables that are correlated with lower levels of self-determination (“lifestyle”, “money”, and “prestige”). Burnout could play a substantial role in the fourth and fifth year [21]. However, our students were enrolled from different academic years, and were not followed over a period of time to be able to establish a trend in motivation types. Therefore, it could be stipulated that some fourth- and fifth-year students had lower levels of self-determination since the beginning of their studies.

Moreover, our study showed that students who were still satisfied with their choice of going to medical school had high autonomous motivation. Sobral et al. established a similar positive correlation between autonomous motivation and students’ intention to continue studies [12].

We expected to find higher levels of self-determination in women, as previously published by Sobral et al. [12] and Kusurkar et al. [13]. However, the difference between genders was not statistically significant in our study. This may be due to the small sample size and the lower response rate compared to the previously mentioned studies.

Higher levels of autonomous motivation were observed in students from USJ who enjoyed their second year training, compared to those who did not. In a study conducted by Ayoub et al. at USJ [14], a positive experience in trainings was shown to increase self-determination in interns. Early contacts with patients through trainings are increasingly studied, and had been shown to be associated with higher levels of autonomous motivation [22–25].

Many studies showed that when autonomous motivation increases, professional outcome and wellbeing of students increase as well [26]. For instance, Kusurkar et al. [4, 27] demonstrated that when the level of self-determination is high, study efforts, deep learning, and academic performance are greater, with a low level of exhaustion. Isik et al. showed that autonomous motivation correlates with higher GPAs [28]. In contrast, another study showed that amotivation is linked to depression and significantly affects medical education outcomes [29]. In both faculties, many measures have already been implemented in order to increase intrinsic motivation. For instance, frequent trainings at the hospital exist, ensuring an early contact with patients and breaking the routine of classes. Additionally, active learning methods are frequently used at the Faculty of Medicine at USJ, such as problem-based learning and small study groups. Soon, a simulation center will also be opened on campus. As for USEK, early contact with patients is ensured through consistent trainings on the field in early years. Although autonomous motivation is the predominating type overall, continuous development of the learning environment may further decrease controlled motivation and amotivation scores, particularly in the fourth and fifth year.

### Implications of our findings

Both faculties have already integrated measures that enhance autonomous motivation in their curricula, such as early contact with patients and problem-based learning. Our results suggest that those measures were successful. However, both faculties could benefit from re-enforcing and enhancing those measures, particularly in the fourth and fifth year, in order to maintain a high level of autonomous motivation among students throughout their program. New measures could also be proposed to stimulate autonomous motivation in students [6, 30]. First of all, students’ activities should be valued since the first year; this may be accomplished by linking theory to practice as much as possible, and would be particularly beneficial in fields of basic sciences, such as biochemistry [10]. Moreover, the perception of autonomy and control should be increased in students, by giving choices throughout the learning process as much as possible [4, 17, 26, 31]. For instance, students should have the possibility to choose their courses, schedules, and training sites. Encouraging participation and strengthening self-efficacy affects performance positively [32]. The perception of competence should also be enhanced, by giving positive and constructive feedbacks [33]. The need for relatedness should be fulfilled as well, through open-mindedness of teachers and other students, discussion, and interest for others. Finally, teachers should choose learning methods that are intrinsically motivating [34, 35], such as problem-based learning, or simulation of clinical situations. They should also use external factors that do not alter intrinsic motivation. This last suggestion is the most difficult to apply, due to the importance of ranking and grading systems, which cannot be easily suppressed. However, the content of examinations could be modified: the whole course should be evaluated, not just restrictive aspects and details that require merely memorization. Finally, early contacts with patients should be maintained and multiplied in both faculties. A positively-perceived learning environment enhances students’ satisfaction, autonomous motivation and behaviors, and leads to greater academic outcomes [33, 36, 37].

### Limitations and strengths

The main limitation of the study was the insufficient number of answers in both faculties, but this is also a valuable information: it may be that the students who did not answer the survey were less intrinsically motivated. A selection bias is present since this study approached two out of seven medical faculties in Lebanon, thus, results cannot be extrapolated to all Lebanese medical students. Information bias might be present since some students might over- or underestimate certain questions/answers. To the best of our knowledge, this study is the first in Lebanon to tackle this topic. It is a first step towards raising awareness among both teachers and students at the faculties of medicine to increase autonomous motivation; this would improve professional outcome, wellbeing, self-satisfaction and performance of medical students.

## Conclusions

This study showed high levels of autonomous motivation in the first five years of medical school. Autonomous motivation was the predominant type in the whole sample. The highest scores of controlled motivation and amotivation were noted in the fourth year. Moreover, high levels of self-determination were seen in students who enjoyed their early contacts with patients through trainings. Actions should be implemented in medical schools to enhance and maintain autonomous motivation, and consequently students’ outcome and health-care quality.

## Data Availability

All data generated or analyzed during this study are not publicly available to maintain the privacy of the individuals’ identities. The dataset supporting the conclusions is available upon request to the corresponding author.

## Abbreviations

AMOT: Amotivation
ERM: External Extrinsic Motivation
MEID: Extrinsic Motivation by Identified Regulation
MEIN: Extrinsic motivation by introjected regulation
MIAC: Intrinsic Motivation for Accomplishment
MICO: Intrinsic Motivation to Knowledge
MIST: Motivation intrinsic to stimulation
SPSS: Statistical Package for Social Sciences
USEK: Holy Spirit University of Kaslik
USJ: Saint-Joseph University of Beirut
